# Accounting for Large Amplitude Protein Deformation during *in Silico* Macromolecular Docking

**DOI:** 10.3390/ijms12021316

**Published:** 2011-02-22

**Authors:** Karine Bastard, Adrien Saladin, Chantal Prévost

**Affiliations:** 1 LABIS, Genoscope, CEA, 2 rue Gaston Cremieux, F-91057 Evry Cedex, France; E-Mail: kbastard@genoscope.cns.fr; 2 MTI, INSERM UMR-M 973, Paris Diderot-Paris 7 University, Bât Lamarck, 35 rue Hélène Brion, F-75205 Paris Cedex 13, France; E-Mail: adrien.saladin@univ-paris-diderot.fr; 3 LBT-UPR 9080 CNRS, IBPC, 13 rue Pierre et Marie Curie, F-75005 Paris, France

**Keywords:** macromolecular docking, flexibility, protein loops and domains

## Abstract

Rapid progress of theoretical methods and computer calculation resources has turned *in silico* methods into a conceivable tool to predict the 3D structure of macromolecular assemblages, starting from the structure of their separate elements. Still, some classes of complexes represent a real challenge for macromolecular docking methods. In these complexes, protein parts like loops or domains undergo large amplitude deformations upon association, thus remodeling the surface accessible to the partner protein or DNA. We discuss the problems linked with managing such rearrangements in docking methods and we review strategies that are presently being explored, as well as their limitations and success.

## 1. Introduction

Macromolecular docking methods aim at predicting the three-dimensional (3D) structure of protein-protein or protein-DNA complexes starting from the coordinates of their components taken in a so-called unbound form. The unbound form may come from the structural databank PDB, in which case it represents the structure of the component alone or in association with another partner. It can also be reconstructed from proteins with homologous sequences and known structure.

In the post-genomic context, docking methods are being developed as a complement to experimental approaches, in order to determine the structure of the macromolecular assemblies that rule the cell life. Knowing the 3D structure of complexes involved in biological processes is necessary for unraveling the mechanism of these processes. In addition, it opens the way to the discovery of new drugs that will enhance or hinder the association. For example, commonly used antibiotics like streptomycin or erythromycin disturb the interactions between ribosome subunits and messenger or transfer RNA of bacteria. In that way protein synthesis is interrupted, which is lethal to the bacteria.

However, a great part of the macromolecular complexes fall out of the reach of experimental methods for high resolution structure resolution, such as X-ray crystallography or nuclear magnetic resonance (NMR). This may be due to their size or to their instability. Besides, the huge number of putative or known complexes involved in biological processes renders necessary the conjunction between structural biology and theoretical tools, provided that the latter reach sufficient levels of accuracy and rapidity. This creates high expectation for the development of macromolecular docking methods.

The field has rapidly progressed since the first docking attempts were performed in 1978 [1], as testified by the results of the CAPRI experiment [2–7]. CAPRI (Critical Assessment of PRotein Interactions, http://www.ebi.ac.uk/msd-srv/capri) challenges modeling groups, involved in the development of macromolecular docking programs, to predict the structure of protein-protein or protein-nucleic acid complexes. The target complexes have experimentally determined but yet unreleased 3D structure. Evaluation is performed on a common basis and the disclosed successes or failures permit to delineate the methodological issues that can be considered solved and those that still need to be improved. The nearly ten years and already twenty three CAPRI rounds have testified increasing quality of the predicted complex structures in spite of the increasing difficulty of the prediction targets.

**Figure 1.**
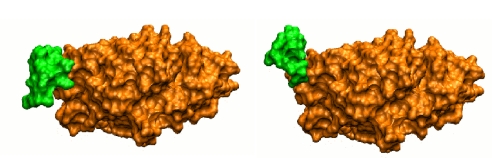
Surface view of actin in its unbound form (left, PDB code 1IJJ) or in complex with deoxyribonuclease I (right, PDB code 1ATN). The two forms differ by the conformation of a short flexible loop, represented in green, which interacts with deoxyribonuclease I in the bound form. The surface accessible to the partner is completely remodeled between the bound and the unbound forms (after Figure 2 of [15]).

Issues related to general macromolecular docking methods or flexible docking have been reviewed in full detail in several highly interesting publications [8–14]. The present contribution focuses on cases where the docking methods still fail to predict the correct geometry of the complex, or at least where potential success cannot be anticipated with certainty. Such cases most frequently occur when the interface of at least one of the partner proteins largely differs between its bound and its unbound forms. This corresponds to situations where the surface that eventually will bind the partner gets remodelled during association (Figure 1), in a process referred to as induced fit. In these cases, neglecting the flexibility during the first phases of the docking simulation can result in the near-native geometry of association not even being sampled. The review first presents the methodological issues associated with taking flexibility into account during docking simulations, followed by an overview of the current knowledge on protein flexibility. Finally, the principal strategies for flexible docking are detailed, with a particular emphasis on the methods aiming at predicting high amplitude conformational changes during macromolecular assembly.

## 2. The Flexible Docking Problem

Typical docking protocols are composed of three ingredients: a representation for the macromolecules (resolution, degrees of freedom for conformational space exploration), a method for searching the available conformational space and a scoring function able to distinguish the correct solution from every other generated structure of the complex.

Searching the possible geometries of binary protein-protein complexes requires the exploration of at least six degrees of freedom, three translations and three rotations. This level of exploration is sufficient as long as the structural modifications of each partner that accompany their association are of limited amplitude (movements of short side-chains, low amplitude deformations of the backbone). The use of low resolution representation with smooth interface, tolerating a certain degree of interpenetration, can then implicitly account for these changes. It is assumed that the protein will eventually perform the small amplitude interface rearrangement that is necessary to optimize the steric and electrostatic complementarity at the interface. As a matter of fact, recent docking programs often follow this assumption in the first phase of a multi-stage strategy [16]. The protein representation can then consist of surface descriptors [17], discretized representations [18,19] or rigid coarse grain models [20,21]. It is accompanied by crude scoring functions, like statistical potential for amino acid-amino acid interaction [21], grid-based estimation of surface contacts [19,22], interaction energy terms limited to electrostatics and van der Waals [20]. At this stage of the docking process, detailed steric complementarity is not necessary for the geometry (position/orientation) of the complex to be considered for further refinement. As a result, while the low resolution search eliminates a huge part of possible geometries of association, it often generates many false positive solutions, *i.e.*, highly ranked predicted structures that are distinct from the near native structure. Recent evaluation showed that for cases with limited overall partner flexibility, when geometries close to the native complex structure pass the first phase, they can be distinguished from the false positive solutions during the second stage, *i.e.*, when small amplitude deformations of the main chain and side chains are explicitly explored [21–27]. In what follows, we will refer to that stage as flexible refinement.

The nature of the problem completely changes when the interface of one component protein gets remodeled during association. The interacting surface may become partially or totally unaccessible to its partner when the protein is in the unbound form. Such cases represent about a quarter of the CAPRI prediction targets and a fifth of non-redundant protein-protein complexes with known unbound form of the interaction partners (benchmark 4.0, [28]). In extreme situations, the spatial arrangement of amino acids characterizing the interface only forms when a loop refolds or when a domain modifies its position or orientation. It is therefore impossible to detect the correct association geometry during a rigid body search phase, even when working at low resolution or with a smooth interface. In addition, even if a geometry close to the native one is retained by chance in spite of incorrect interface, it cannot be expected to be improved during the refinement phase. As already mentioned, the refinement phase generally introduces spatial limitation for the relative position/orientation of the two partners, while the route towards the correct geometry of association would necessitate the separation of the partners to allow loop refolding or domain repositioning. Such cases can only be solved if flexibility is considered from the beginning of the docking process.

This formally transforms a search problem with six degrees of freedom into a search problem with 3N degrees of freedom, where N represents the total number of atoms in the system. The presently available computer resources can account for such high number of degrees of freedom, for example during numerical MD simulations, but the conformational space explored by these simulations is limited by the simulation time. Since the largest amplitude internal moves are also those that take more time to be sampled, such methods appear unsuitable to the docking search problem, which requires high velocity. This makes it necessary to reduce the search dimensionality. Section 3 displays the strategies that have been developed to address this challenge, based on the growing knowledge of the characteristics of protein flexibility. It is useful to present some elements of this knowledge for the understanding of flexible search strategies, and this is done in Section 2.2.

### 2.1. Flexible Refinement

What characterizes the flexible refinement stage is that it deals with already formed complex structures resulting from the search stage of docking. The process then allows only small spatial adjustments of the global geometry in terms of the six rigid body degrees of freedom. It concentrates on the explicit account of internal degrees of freedom that are responsible for surface adjustments. The spatial limitation of the search together with the fact that the number of refined structures is reduced with respect to the first phase of exhaustive search make it possible to introduce some degree of internal flexibility in the exploration. The task remains however difficult. Searching the side chain conformations that optimize the interface is computationally expensive for protein-protein complexes, where the interfaces of both partners need to be simultaneously optimized in combination with small amplitude rigid body adjustments. Side chain rotamer libraries, gathering the conformations most frequently found in known protein structures, can be used with benefit in the process [29]. While the conformation of particular side chains in the bound form does not necessarily coincide with a rotamer, the use of discrete side chain representations can be justified in preliminary search phases. The group of Camacho has shown for 39 protein test cases that the bound conformation of key side chains is frequently visited during molecular dynamics simulations of the free form of the protein. [30,31]. Sophisticated algorithms have been developed for predicting the rotamer combinations that best fit a fixed backbone. However, when searching rotamer libraries in conjunction with backbone repositioning in docking programs, it is convenient to use Monte Carlo simulations, which are also well adapted to directly sampling the dihedral angles in internal variable representation [21,22,32]. The use of molecular dynamics (MD) simulation for side chain final refinement, sometimes with explicit solvent representation, is now commonly encountered due to increased computational facilities.

Protein backbone refinement has been introduced only recently in the systematic docking programs. It is however at the heart of the information-driven docking program HADDOCK developed by the group of Bonvin [33,34]. This program initially performs a rough positioning of the two partners relative to each other, based on the satisfaction of a set of interaction restraints derived from experimental data (NMR, biochemical data). A particularity of the method is that the restraints are not attributed to specific residue pairs, with each residue belonging to a different partner, but rather that they concern pre-defined groups of residues for each partner, hence the term “ambiguous restraints”. After these restraints are satisfied, the protocol can concentrate on the refinement stage, which is performed in two steps, first using MD simulations in internal coordinates with side chain and backbone flexibility limited to the interface regions, then using cartesian coordinate MD simulations in explicit solvent environment.

With the progress of computational performance and algorithmic development, methods that combine side chain conformational search, ligand repositioning and main chain limited structural adjustment are emerging [24,35,36]. Erlich *et al.* [37] have emphasized the necessity of simultaneously taking into account these three levels of conformational search or adjustment to achieve successful docking refinement.

### 2.2. Characteristics of Protein Flexibility

Although proteins are generally characterized by well defined tertiary structures, they undergo structural fluctuations at biologically relevant temperatures. Part of these movements can be described by high frequency, small amplitude fluctuations of the atoms around their equilibrium position (harmonic fluctuations). However, a large part of the movements (up to 80%) can be accounted for by a few modes of deformation that involve concerted atomic motions. Such directions of deformation can be accessed by several methods, including normal mode analysis or principal component analysis of structural ensembles [38]. For example, Dobbins *et al.* [39] have shown that in 35% of the proteins they studied, the direction of the observed conformational change could be correctly described by a single low-frequency normal mode. The ensembles may be composed of MD snapshots or structures obtained in different conditions and environments by X-ray crystallography or NMR.

Large amplitude fluctuations of protein structures often present biological importance. They have been shown in various occasions to play a role in catalytic reactions, in molecular assembly or in specific ligand recognition. Concerted movements can present a strong harmonic character, generally with low frequency fluctuations. In this case, they can be addressed by simple analytic functions during docking calculations, as shown in the next section. Alternatively, when the concerted movements involve main chain refolding, the protein structure explores various conformational substates separated by energy barriers with small to large amplitudes. Generating and exploring these substates then becomes highly time consuming, which is not compatible with a docking search process. In those cases, it is useful to consider another characteristic of protein flexibility, which is its anisotropy. At the residue level, protein display large variations in fluctuation amplitudes [40,41]. Interestingly, Sacquin-Mora and Lavery [42,43] have shown that among the residues that present high degrees of rigidity, several are key residues involved in catalytic activity or in the stabilization of the overall 3D structure. Rigid residues can also be found at the frontier between domains or at the extremity of loops, where they constitute hinge points between protein regions. As a matter of fact, interface remodeling in interacting protein most often results from the movement of loops or domains that move independently from an almost rigid protein core. For the proteins that fall in this category, it is possible to restrain the search to the flexible regions during the docking search phase, as shown in the next section. Several web servers are devoted to the detection of flexible protein segments that may be involved in interface remodeling during docking. ElNemo [38] (http://www.igs.cnrs-mrs.fr/elnemo/) and HingeProt [44] (http://bioinfo3d.cs.tau.ac.il/HingeProt/hingeprot.html) are based on the analysis of low frequency normal modes calculated from elastic networks, while ProFlex [45] (http://www.bch.msu.edu/kuhn/software/proflex/) relies on a three-dimensional constraint counting algorithm. StoneHinge [46] is a consensus method between the two approaches.

The two types of deformation that have been described above can be put in correspondence with two complementary views of protein structural adjustments upon interaction. In the induced-fit interpretation, the protein modifies its internal conformation due to its exposition to the field of its partner [47]. In the structural ensemble interpretation, all conformational substates a protein can occupy coexist in solution, and the partner will select the substate that allows optimal surface fit [48].

## 3. Methods for Flexible Docking Search

Based on the protein deformation characteristics described above and on the different views of the docking process, several strategies have been developed to deal with flexibility from the beginning of the docking process. In what follows, the term “continuous approach” will refer to strategies where the molecule gradually deforms to fit its partner. To the contrary “discrete approaches” refer to strategies where each molecule selects the best fitting conformation among ensembles of substates (or conformers) that represent its partner.

### 3.1. Continuous Approaches

#### 3.1.1. Soft Mode Relaxation

A first strategy is to introduce concerted movements as additional degrees of freedom during the search phase. As explained above, introducing full internal flexibility during the search would be intractable, given the objective of rapidity and efficiency. However, when the movement can be described by harmonic approximation, the largest amplitude conformational changes only require a few selected modes of deformation. Restricting the search to this few modes permits to reduce the dimensionality of the problem. Indeed, the group of Zacharias has shown that the use of only three modes could drastically improve the docking result between RNAse A and its porcine inhibitor [49]. In that work, exploration is performed using the docking program ATTRACT with reduced protein representation [20]. The program bases the search on a multi-minimization algorithm. In the rigid body version, the interaction energy between a mobile ligand (the smallest of the two partners) and a fixed receptor is optimized with respect to six degrees of freedom. In the flexible version, the selected collective variables are added as new degrees of freedom, with an associated potential energy *E*(*x*) *α* (*x* − *x*_0_)^4^, where *E*(*x*) is the energetic penalty term associated to the deviation (*x* − *x*_0_) of the collective variable *x* with respect to its starting value *x*_0_. In this expression, the power four allows confinement of the collective variable to values where it retains its harmonic character, thus avoiding the generation of unphysical structural deformations.

The soft mode relaxation method therefore offers the potentiality to reduce the computational complexity at low calculation cost.

### 3.2. Discrete Approaches

#### 3.2.1. Cross-Docking

Several groups have investigated an approach of flexibility where predefined ensembles of conformers are cross-docked, *i.e.*, each conformer of one partner is successively docked with each conformer of the other partner. The ensembles can be constructed from conformations available in databanks, for example as a result of NMR structure determination. More generally, they can be constructed from essential or enhanced MD simulations [50–53]. The numerous complex structures resulting from cross docking are then clusterized and ranked. All reported cross-docking experiments generated clusters of solutions closer to the correct complex structure than the equivalent rigid body docking starting from the unbound form. However, these structures did not necessarily rank among the 10 clusters with best scores and many false positive were also generated. Due to their calculation cost, cross-docking experiments were initially used to investigate the thermodynamics and kinetic driving forces of the protein-protein assembly process [50]. The impressive progress in docking program velocity permits to conceive cross-docking as a routine process for flexible docking [54].

#### 3.2.2. Interface Remodeling

The particularities of protein flexibility permit to more specifically restrain the search to movements of flexible fragments, either loops or domains, while representing the rest of the protein as a rigid body with smooth interface. In addition to sparing calculation time, spatial limitation permits to enhance the exploration of the flexible fragments and to access totally anharmonic moves such as loop refolding or domain rearrangement. This implies preliminary detection of the flexible fragments, as already reported in Section 2.2. Since in the ensemble-based approaches the loop conformers are pre-generated, large exploration methods like robotics [55] or alphabet-based analysis [56] can be called in the preparation phase, particularly in the case of long loops (more than 12 amino acids) that remain a real challenge for modelling methods. The particular problem of protein loop structure determination is recurrent in the molecular modeling literature. Flexible loops are common at protein surfaces and the sequence difference between two homologous proteins often concentrates on such fragments, in association with variations in function. Useful articles reporting on the loop prediction methods can be found in references [57,58]. Note that the major part of these methods aim at predicting only the lowest energy conformations, while accounting for loop conformational changes requires the access to every possible conformational substate.

#### 3.2.3. Multi-Copy/Mean Field Approach

Bastard *et al*. [32,59] have shown that when using loop ensembles that cover the whole volume accessible to a flexible loop, it is possible to improve the prediction of both the relative arrangement of the partners and the loop geometry through a unique docking simulation. For this purpose, the flexible loop was taken into account via the mean field theory: each substate (copy) is attributed a weight which is iteratively adjusted during the docking process, depending on the interaction energy between the copy and the partner of association. In that way, the best interacting copies are attributed the highest weight, which in turn increases their influence in the docking process.

The method has been successfully coupled to two search methods: Monte Carlo simulations and minimization. Monte Carlo search was used for protein-DNA docking in all atom representation with the MC2 program [32]. In this study featuring a paired domain and its target oligonucleotide, the copy weights of a copy ensemble comprising 66 loop conformers were readjusted at the end of each of five runs of a Monte Carlo simulated annealing cycle, in function of the mean interaction energy observed during that cycle. In spite of the high amplitude allowed for loop movements, this first application of the multi-copy/mean field method more specifically resorts to refinement docking: the search space was restrained to the vicinity of the flexible loop, the search was performed in atomic representation and resulted in the reproduction of the atomic details of the native interface. The minimization-based ATTRACT application of the multi-copy/mean field algorithm illustrates the introduction of flexibility from the beginning of the search phase of a systematic docking procedure. For this reason, we will rather center the discussion on that application. In this case, the number of copies representing the flexible fragment is reduced due to the coarse-grained representation. The copy weights directly influence the energy minimization process by entering in the calculation of the energy derivative [59]. The use of the multi-copy/mean field method for systematic docking was first tested on a set of eight protein/protein complexes where one or two loops present important conformational changes between the bound and the unbound form of one partner [59]. When the correct loop conformation was present among the copy ensemble, the correct arrangement of the partners and the correct loop conformation could be unambiguously predicted. When it was not, the ranking of solutions close to the complex geometry was improved in all cases, together with the population of corresponding clusters of solutions.

**Figure 2.**
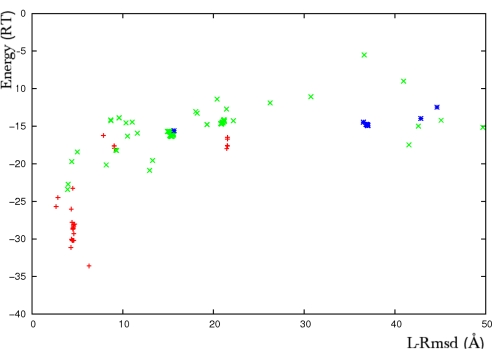
Results of an ATTRACT flexible docking simulation of the CDK2-cyclin complex (PDB code 1FIN). Plot of the interaction energy (RT units) *versus* the ligand root mean square deviation (rmsd, Å). The rmsd is calculated on the *Cα* atoms of the predicted ligand with respect to its structure in the crystallographic complex. The flexible parts represent near 30% of the whole receptor CDK2. They are formed by an 80 amino-acid domain with internally articulated movement (deviation of 4.4Å between the *Cα* atoms of the unbound and bound forms) and a 9 amino-acid loop that refolds upon association (13.2Å deviation). The multi-copy/mean field algorithm was applied to two two-copy ensembles formed by the bound and the unbound forms of the two flexible fragments. The rigid protein core was taken in its unbound form. Red crosses represent predicted solutions where both the domain and the helix were found in the bound form, green crosses correspond to solutions where at least one flexible part was predicted in the unbound form and blue stars correspond to predictions where both parts were predicted in the unbound form.

**Figure 3.**
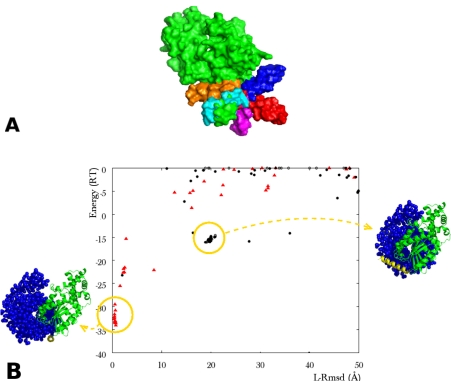
(**A**) Surface view of the *α* subunit of transducin (green), with ten helix copies. The copies (various colors) occupy the space available to the crystal helix (green); (**B**)Plot of the interaction energy (RT units) versus ligand rmsd (Å) reporting the results of an ATTRACT docking simulation between the *α* and the *β*, *γ* subunits of transducin (PDB code 1GOT). The rmsd is calculated in the same way as reported in Figure 2. The flexible parts are a 21 amino acid *α* helix, unstructured in the unbound form, and a 17 residue loop (rmsd apo/holo on *Cα*: 6.1Å). The helix was represented by a set of ten conformers, including the crystal helix. The loop was represented by a two-copy ensemble (bound, unbound). In order to separate out the effect of multi-copy representation, the rigid protein core was taken in its bound form in this simulation. The red triangles represent the solutions where the bound forms of both the helix and the loop were selected by ATTRACT. Yellow circles indicate the location of two clusters of solutions. The corresponding geometries of association are represented in blue for the *β*, *γ* subunits of transducin (van der Waals representation), in green for the rigid core and in yellow for the flexible helix of *α* transducin (ribbon representation).

Several issues have been further investigated to evaluate the possible use of the method in general blind docking predictions (unpublished results). It was found that the method can successfully apply to systems with more than one flexible part (see Figures 2 and 3), to systems where the flexible fragment occupies an important fraction of the structure (30% for CDK2-cyclin complex, PDB code 1FIN) or where it can span a large accessible volume (*α*-*β*, *γ* subunits of transducin, PDB code 1GOT). For both cases 1FIN (Figure 2) and 1GOT (Figure 3), when the correct conformation was present in the copy ensemble, the algorithm ranked first solutions close to the native complex structure and correctly selected the bound forms for both the loop and the domain. Figure 3B shows two structures corresponding to two different clusters of solutions, a solution close to the experimental complex (left) and an alternative positioning of the ligand, favored by a different orientation of the helix in yellow (right). The energy score clearly discriminates the near-native structures from other potential solutions.

**Figure 4.**
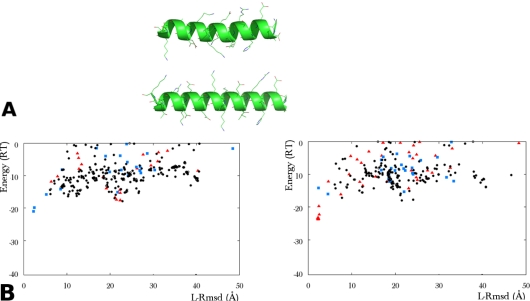
Tolerance to structural inaccuracies in the helix representation of 1GOT by a multi-copy conformer ensemble. (textbfA) Ribbon representation of the crystal helix (top) and an *ab initio* constructed helix (bottom). The rmsd between the generic helix and the crystal helix is 0.7Å for the *Cα* atoms, 2.1Å for all atoms. (textbfB) Results of two ATTRACT flexible docking simulations with an ensemble of nine helix copies. The rigid protein core is in unbound form. (left) The copies were obtained by superposing *ab initio* constructed helices on each of the ten conformers used in the simulation of Figure 3, with the exception of the crystal helix. Red triangles indicate the predicted structures where the copy closest to the crystal helix (5Å rmsd on *Cα* atoms) was selected. (right) Same simulation as shown left, except that the copy deviating by 5Å was replaced by an *ab initio* conformation with *Cα* atoms deviating by 1Å from the crystal. The predicted structures where this copy was selected are represented by red triangles. In both simulations, blue squares correspond to the selection of a copy deviating by 21Å rmsd from the crystal helix.

Tolerance of the method to structural inaccuracies was investigated in the case of the flexible helix of 1GOT. When the crystal helix position was absent from the conformer ensemble, and *ab initio* constructed helices were superposed on each of the nine remaining copies, the closest copy (5Å deviation on *Cα* atoms) did not favor the correct positioning of the ligand. In the simulation displayed in Figure 4B (left), the best ranking solutions present a correct positioning, but with a selected helix conformation deviating by 21Å from the crystal helix. When the closest copy deviated by 1Å from the crystal instead of 5Å, it displaced the 21Å copy and was selected in near-native solutions with improved interaction energy (Figure 4B, right).

Combined together, these results raise two issues: the first one concerns the quality of the conformer ensemble (spatial density and extent) that is necessary to reach an accurate prediction. To our knowledge, this issue has not been extensively investigated in the cross-docking studies. It has been addressed in the case of the multi-copy/mean field approach of loop flexibility by Loriot *et al.*. [60]. In this work, the spatial density of the conformers in the multi-copy ensemble was optimized using a greedy algorithm, in order to span the whole space available to the loop with the minimum number of conformers. Spatial dispersion of the conformers is an important factor in this method since local conformer accumulation may bias the mean field resulting from the copy ensemble. In the four tested protein-protein cases, it was found that total coverage of the available space could be achieved using ten to twenty copies.

**Figure 5.**
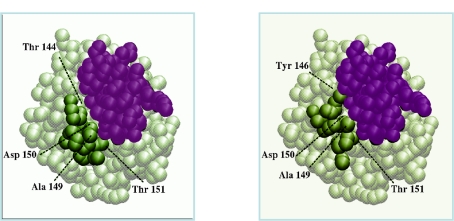
Interface reconstitution resulting from the flexible docking of chymotrypsinogen to its inhibitor (PDB code 1CGI). In this simulation, the flexible loop was modelled by a nine-copy ensemble where the experimental backbone fold was not represented. (left) Coarse grain representation of the crystal complex, with the inhibitor in purple, the rigid part of chymotrypsinogen in grey and the flexible loop in green; (right) Best predicted complex (same color code). The inhibitor deviates from its experimental position by 1.3Å. The loop rmsd on *Cα* atoms between the two views is 4.4Å. Alanine 149, asparagine 150, and threonine 151 of the predicted loop interact with the inhibitor similarly than in the crystal complex. Threonine 144 replaces tyrosine 146 of the bound loop (after Figure 4 of [59]).

The second issue addresses the methodological problem of interface “reconstitution” from two or more structural elements. As illustrated in Figure 3, highly ranked solutions resulting from the multi-copy/mean field approach generally present a continuous, yet composite interface, with one part belonging to the rigid portion of the flexible protein and the complement to the flexible part. The problem may therefore be put in terms of the generation of a complementary fraction of the rigid interface part, from the refolding/positioning of the flexible part. Interestingly, correct position/orientation of the ligand could be obtained for highly ranked predicted structures even for limited spatial density of the conformer ensemble, which also limits the probability to include a conformer close to the experimental structure in the copy ensemble. In one interesting case [59], the interface between a flexible loop and the association partner could be almost entirely reconstituted from a misfolded conformer, which positioned key loop residues at favorable positions (Figure 5). More often, it was found that in the tested cases, only few residues from the flexible parts needed to interact with the docking partner for the resulting complex to increase its interaction energy above the background noise (results not shown). While the generalization of this observation remains to be established, the notion of composite interface rebuilding appears to constitute an important component of the success of two flexible docking search methods presented below, which deal with large amplitude interface flexibility.

#### 3.2.4. Multi-Component Docking

When the protein deformation is composed of hinge motions between two or more domains, it is possible to artificially split the flexible domains and to separately assemble them with the partner of association within multibody docking algorithms [61,62]. While the approach developed by the Eisenstein group was a multibody, multistage approach (docking D1 to L, D2 to L then D1 to D2-L and D2 to D1-L, where D1, D2 are two domains of the receptor moving independently as rigid bodies, and L is the ligand), the groups of Nussinov and Wolfson have developed a high performance algorithm, built on geometric hashing techniques, to generate topologically feasible and energetically favorable complexes with composite interface, starting from the results of an initial set of two-body docking simulations [61,63,64]. The algorithm was initially developed to performing multi-macromolecular docking (CombDock [63,65], which requires the resolution of a highly complex combinatorial search problem. The only difference with flexible hinge domain docking is that specific restraints were added to the process to ensure that the split protein can be reconstructed at the end of the process. A consequence is that it can be applied to proteins where the arrangement of several domains is modified upon association. The high velocity of the docking search method, based on the comparison of surface descriptors for each partner of association [17], makes it possible to routinely run flexible docking simulations involving hinge-type domain motions [61]. The adaptation of multi-macromolecular docking to hinge domain flexible docking via the introduction of restraints appears particularly well adapted to data-driven type docking methods like HADDOCK [66]. Since multi-macromolecular docking facility was recently introduced into the HADDOCK web server [67], it is now possible to perform hinge domain flexible docking on the HADDOCK Web server (http://hadock.chem.uu.nl).

An interesting observation from multi-component docking simulations is that the partial two-body interfaces that are selected as component of the final system interface often rank poorly in the initial two-body docking simulations [63,68]. Similarly to what was observed for the flexible loops, these partial interfaces need to be reinforced by complementary interactions in order for the correct assembly to be distinguished from alternative solutions.

##### 3.2.4.1. Interactive Loop Reconstruction

Recently, an astute solution was included in the RosettaDock multi-scale Monte Carlo docking program to account for high amplitude loop remodeling during docking processes [24]. In this approach, the flexible loop is artificially removed from the protein and is rebuilt during the docking process with RosettaDock, first at low resolution during the early docking stages and then at higher resolution during refinement search. The reconstruction involves robotic algorithms for loop closure together with Monte Carlo and Monte Carlo minimization moves for structure sampling. Recently, Mandell and Kortemme proposed an analytical loop reconstruction algorithm, also based on robotic techniques, for precise loop reconstruction at atomic resolution [69]. Optimally fitting long loops to a DNA surface during docking could also be achieved using interactive molecular dynamics simulation [70]. Again, the efficiency of loop reconstruction methods is strongly dependent on the existence of a composite interface, partly formed from the rigid protein core and partly from the flexible loop.

## 4. Conclusions

**Figure 6.**
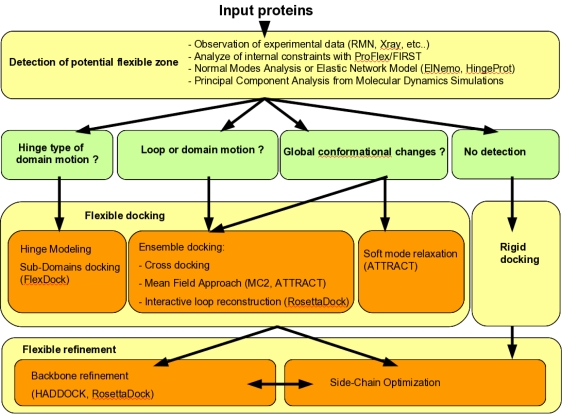
Workflow of strategies that decide which flexible docking methods have to be used.

Taking protein flexibility into account in docking protocols is a highly challenging methodological problem, where combined levels of search and refinement need to be integrated, from low resolution and high amplitude movements to atomic relaxation of side chains and backbones. Innovative solutions have been proposed during recent years at each of these levels, which announce in the near future the release of fully flexible docking programs capable of handling every type of flexibility, for example solving all the “difficult” docking cases of the protein-protein benchmark. Such programs may consist of a modular workflow with automatic or user-input decision steps concerning the level of flexibility (local or global), the method to be employed, the pre-generation of conformer ensembles. An example of such workflow is presented in Figure 6. Further understanding of the mechanism of protein deformations, as well as ongoing investigations on the characteristics of protein-protein interfaces, will be useful to improve the efficiency of flexible docking algorithms.
